# Modulation of Inflamed Synovium Improves Migration of Mesenchymal Stromal Cells in Vitro Through Anti-Inflammatory Macrophages

**DOI:** 10.1177/19476035221085136

**Published:** 2022-03-19

**Authors:** Marinus A. Wesdorp, Yvonne M. Bastiaansen-Jenniskens, Serdar Capar, Jan A.N. Verhaar, R. Narcisi, Gerjo J.V.M. Van Osch

**Affiliations:** 1 Department of Orthopaedics and Sports Medicine, Erasmus MC, Rotterdam, The Netherlands; 2 Department of Otorhinolaryngology, Erasmus MC, Rotterdam, The Netherlands; 3 Department of Biomechanical Engineering, Faculty of Mechanical, Maritime, and Materials Engineering, Delft University of Technology, Delft, The Netherlands

**Keywords:** macrophages, synovial inflammation, MSC, cartilage repair

## Abstract

**Objective:**

Inflammation is known to negatively affect cartilage repair. However, it is unclear how inflammation influences the migration of mesenchymal stromal cells (MSCs) from the underlying bone marrow into the defect. We therefore aimed to investigate how synovial inflammation influences MSC migration, and whether modulation of inflammation with triamcinolone acetonide (TAA) may influence migration.

**Design:**

Inflamed human osteoarthritic synovium, M(IFNγ+TNFα) pro-inflammatory macrophages, M(IL4) repair macrophages, M(IL10) anti-inflammatory macrophages, or synovial fibroblasts were cultured with/without TAA. Conditioned medium (CM) was harvested after 24 hours, and the effect on MSC migration was studied using a Boyden chamber assay. Inflammation was evaluated with gene expression and flow cytometry analysis.

**Results:**

Synovium CM increased MSC migration. Modulation of synovial inflammation with TAA further increased migration 1.5-fold (*P* < 0.01). TAA significantly decreased *TNFA*, *IL1B*, and *IL6* gene expression in synovium explants and increased *CD163*, a gene associated with anti-inflammatory macrophages. TAA treatment decreased the percentage of CD14+/CD80+ and CD14+/CD86+ pro-inflammatory macrophages and increased the percentage of CD14+/CD163+ anti-inflammatory macrophages in synovium explants. Interestingly, MSC migration was specifically enhanced by medium conditioned by M(IL4) macrophages and by M(IL10) macrophages treated with TAA, and unaffected by CM from M(IFNγ+TNFα) macrophages and synovial fibroblasts.

**Conclusion:**

Macrophages secrete factors that stimulate the migration of MSCs. Modulation with TAA increased specifically the ability of anti-inflammatory macrophages to stimulate migration, indicating that they play an important role in secreting factors to attract MSCs. Modulating inflammation and thereby improving migration could be used in approaches based on endogenous repair of full-thickness cartilage defects.

## Introduction

Articular cartilage defects pose a significant clinical challenge in the orthopedic field. Cartilage defects of the joint are common and if left untreated can lead to the development of early-onset osteoarthritis (OA).^1,2^ Cartilage defects have a limited regenerative capacity, as the articular cartilage is avascular,^3^ combined with an impaired migration capacity of cartilage cells through the dense extracellular matrix.^4,5^ Therapies involving bone marrow stimulation techniques can partially overcome this problem by creating access to the bone marrow reservoir. Bone marrow–derived stromal cells (MSCs) are considered as a promising cell type for the repair of damaged cartilage due to their chondrogenic differentiation potential.^6,7^ However, the migration of MSCs from the underlying bone marrow and the chondrogenic differentiation will often have to occur in a chronic inflamed joint environment.

Joint inflammation may inhibit successful cartilage defect repair and without successful repair cartilage defects can eventually lead to the development of OA.^8,9^ Inflammatory cytokines are produced by the synovium,^10^ the highly vascularized mucosal lining of the knee joint that is responsible for the production of synovial fluid that lubricates and nourishes the cartilage.^10,11^ The synovium contains macrophages that are key players in inflammation and wound healing in injured or osteoarthritic joints.^12^ Depending on the stimuli they receive from their microenvironment, macrophages can polarize to distinct phenotypes, referred to as pro-inflammatory macrophages, repair macrophages, and anti-inflammatory macrophages.^13^ Pro-inflammatory macrophages inhibit the formation of cartilage *in vitro*, while anti-inflammatory macrophages or repair macrophages stimulate the formation of cartilage *in vitro.*^14^ A potential therapeutic option could be to inhibit or reduce inflammation to improve cartilage repair. However, it is unknown what the effects of modulating inflammation would be on the migration of MSCs toward the defect site in an earlier phase of the repair process.

MSC migration is an essential step in the repair process, because MSCs first need to migrate into the defect before starting the process of cartilage formation.^15^ Clinically, migration of MSCs into the defect can be enabled by techniques such as microfracture.^16^ With this technique, a direct connection is created between the cartilage defect and the underlying bone marrow that contains MSCs. This connects MSCs not only with the defect site, but also with the inflamed intra-articular joint environment. Inflammation is known to play an important role in the migration of MSCs toward an injured site.^17,18^ Studies have shown that the migration of MSCs to injured sites is regulated by chemokines, cytokines, and growth factors. These factors have been widely investigated and overall have a stimulatory effect on the migration capacity of MSCs.^19^ However, in higher concentrations these factors inhibit MSC migration.^20^ In the case of joint inflammation, however, the situation is even more complex due to the presence of a “cocktail” of pro-inflammatory, but also antagonistic anti-inflammatory factors. Therefore, migration cannot be attributed to one specific factor and it is unknown what reducing joint inflammation would mean for MSC migration.

Anti-inflammatory drugs such as triamcinolone acetonide (TAA) are used to reduce inflammation and reduce the symptoms of OA.^21^ Over the years there has been an increasing interest in the use of anti-inflammatory drugs immediately after various intra-articular pathologies to reduce joint inflammation, synovitis, and longer-term post-traumatic OA.^22,23^ The effect of anti-inflammatory medication on the synovium, cartilage, and bone has been studied extensively.^24,25^ However, the effect it has on the migration of repair cells remains unclear. In the current study, we investigated how MSC migration is influenced by conditioned medium (CM) produced by inflamed synovium focusing on the influence of modulating inflammation with glucocorticoid TAA. We hypothesized that the anti-inflammatory effects of TAA treatment would enhance MSC migration. Furthermore, we investigated which cell types present in the synovium might be responsible for the effect.

## Materials and Methods

### Generation of CM from Synovium and Synovial Fibroblasts

Synovial tissue was obtained from 9 patients with knee OA (4 female, 5 male, 63 ± 8.3 years old) undergoing total knee arthroplasty (Suppl. Table S1). The synovium samples were obtained with implicit consent as waste material from patients undergoing knee replacement surgery (approved by the local ethical committee; MEC2004-322). The patients had the right to refuse as stated by the guidelines of the Dutch Federation of Biomedical Scientific Societies (www.federa.org). Synovium tissue was washed 2 times with 0.9% NaCl (Sigma-Aldrich, St. Louis, USA), separated from the underlying fat, cut into small pieces of approximately 50 mg wet weight. Dulbecco’s Modified Eagle Medium, low glucose (DMEM-LG; Gibco, Carlsbad, USA) supplemented with 1% Insulin-Transferrin-Selenium (ITS Premix, Corning, Tewksbury, USA), 50 mg/mL gentamicin (Gibco), 1.5 mg/mL amphotericin B (Fungizone; Gibco) were used to culture the synovial tissue for 24 hours, using 1 mL of media per 200 mg of tissue.

**Figure 1.**
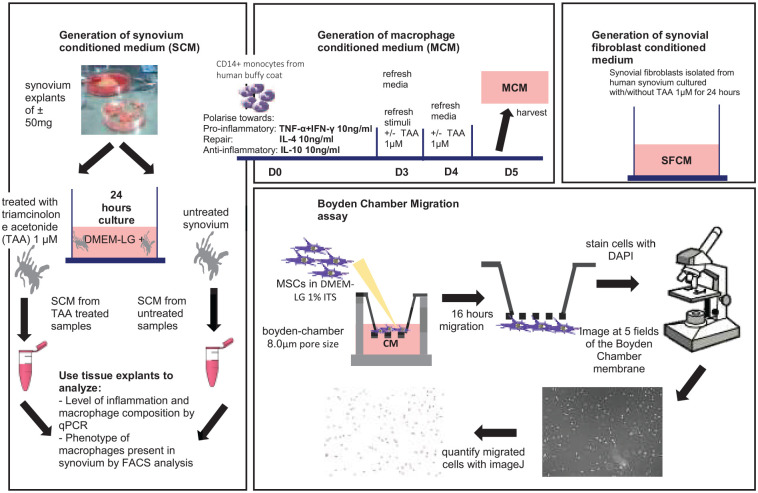
Experimental design on generation of conditioned media and their use in MSC migration assay. Conditioned medium from 9 synovium donors of which 6 were treated with TAA and DMSO as vehicle control, 4 monocyte donors and 3 synovial fibroblast donors were generated. MSC = bone marrow-derived mesenchymal stem/stromal cell; TAA = triamcionolone acetonide; DMSO = dimethyl sulfoxide; DMEM-LG = Dulbecco’s Modified Eagle Medium, low glucose; FACS = fluorescence activated cell sorting; SFCM = synovial fibroblast conditioned medium; MSCs = mesenchymal stromal cells; CM = conditioned medium; DAPI = 4′,6-diamidino-2-phenylindole.

Explants were cultured with or without 1 µM of the anti-inflammatory drug TAA) (Sigma-Aldrich) at 37°C. The concentration for TAA was based on previous studies we performed with human synovium.^26,27^ Prior to starting our study, we used 2 dosages of TAA (1 and 10 µM) and confirmed that they were both effective (data not shown). We selected to lowest effective dose of 1 µM. Dimethyl sulfoxide (DMSO; Sigma-Aldrich) was used as vehicle control for TAA and the final DMSO concentration in the cultures was 0.01%. After 24 hours, synovium was harvested, and either snap-frozen in liquid nitrogen for later RNA isolation or digested for flow cytometric analysis. Also, the CM was harvested, centrifuged at 300*g* for 8 minutes to remove the debris, and stored at −80°C for subsequent Boyden chamber migration assay (**Fig. 1**). Unconditioned control medium was generated by incubation of DMEM-LG with 1% ITS, 50 mg/mL gentamicin and 1.5 mg/mL amphotericin B for 24 hours at 37°C, centrifuged at 300*g* for 8 minutes, and stored at −80°C.

Fibroblast-like synoviocytes were isolated from the synovium as described before.^28^ Passage 3 cells were seeded at a density of 50,000 cells/cm^2^ in culture medium. After attachment overnight, the culture medium was removed, cells were washed 3 times with saline, and fresh medium was added containing DMEM-LG (Gibco), 1% ITS (Corning), 50 mg/mL gentamicin (Gibco), 1.5 mg/mL amphotericin B (Gibco). The fibroblast-like synoviocytes were cultured for 24 hours in the presence of 1 µM TAA (Sigma-Aldrich) or 0.01% DMSO as vehicle control. After 24 hours the CM was harvested, centrifuged for 8 minutes at 300*g* and stored at −80°C until further use in the Boyden chamber migration assay (**Fig. 1**).

### MSC Migration Assay

Human MSCs (1 female, 2 male, 54.3 ± 15.7 years old) were isolated by using bone marrow aspirates from patients with hip OA, undergoing total hip arthroplasty after written informed consent and with approval by the local ethical committee (Erasmus MC University Medical Center, The Netherlands, MEC 2015-644 and Albert Schweitzer Hospital: protocol 2011.07) (Suppl. Table S2). Nucleated cells from the heparinized bone marrow aspirates were seeded at the density of 300,000 to 600,000 cells/cm^2^ in Minimum Essential Medium-Alpha expansion medium (α-MEM; Gibco™) supplemented with 10% heat-inactivated fetal calf serum (FCS; Lonza), 50 mg/mL gentamicin (Gibco), 1.5 mg/mL amphotericin B (Fungizone; Gibco), 1 ng/mL fibroblast growth factor 2 (FGF2; R&D Systems), and 25 μg/mL ascorbic acid-2-phosphate (Sigma-Aldrich). After 24 hours, nonadherent cells were washed away with phosphate-buffered saline (PBS) containing 2% FCS and adherent cells were further expanded in expansion medium, trypsinized at subconfluence and seeded again at a density of 2,300 cells/cm^2^. Cells were refreshed twice a week and trypsinized in case of subconfluency and passage-3 (P3) cells were used for the migration assays. Viability assessed with trypan blue using a hemocytometer revealed >95% of the MSCs were viable.

Migration assays were performed using a 24-well Boyden chamber setup with cell culture inserts of 8.0 µm pore size (Corning, Tewksbury, USA). For each cell culture insert 15,000 MSCs were used, suspended in 200 µL DMEM-LG (Gibco, Carlsbad, USA) supplemented with 1% ITS, 50 mg/mL gentamicin (Gibco), 1.5 mg/mL amphotericin B (Fungizone; Gibco) and seeded on top of the insert membrane. CM was thawed on ice and mixed prior to use with a predetermined volume of DMEM-LG (Gibco, Carlsbad, USA) medium containing 1% ITS Premix (Corning, Tewksbury, USA), 50 mg/mL gentamicin (Gibco) and 1.5 mg/mL amphotericin B (Gibco) corresponding to a percentage of CM in the final medium (**Fig. 1**). In total, 600 µL of medium containing CM was added in the lower chamber. After 16 hours of incubation in a humidified incubator of 37°C and 5% CO_2_, transwell inserts were carefully removed from the plate as well as the medium inside the insert. The inserts were then washed with PBS, fixed by 4% formaldehyde for 20 minutes and the nonmigrated cells from the upper part of the membrane were removed with a cotton swab. The cells that had migrated to the other side of the membrane were immediately stained with 4′,6-diamidino-2-phenylindole (DAPI; ThermoFisher). Images of the migrated cells on the lower part of the membrane were taken at 5 nonoverlapping fields of 0.017 cm^2^ from the center, top, bottom, right, and left parts of the membrane with a fluorescent microscope (Leica SP5). For each image, the cell number was counted using Image-J software utilizing the analyze particles tool. The cell numbers of the 5 fields covered 25% of the total area of the membrane. The cell numbers of each field were added and this number was converted to match the entire area of the Boyden chamber membrane (0.33 cm^2^). The cell number of each membrane was normalized to the average of the number of cells that migrated in the control conditions. The average of the unstimulated control conditions was set to 1. Since we had 3 to 5 samples of unstimulated conditions for each experiment, we also normalized these unstimulated control conditions for the average and thus show the variation in this condition.

### RNA Isolation and Quantitative Real-Time PCR

Snap-frozen synovium explants were pulverized with a Mikro Dismembrator (Braun Biotech International GmbH, Melsungen, Germany) at 3,000 rpm for 30 seconds. The samples were resuspended in 1 mL RNA-Bee (TelTest, Friendswood, Texas, USA). Chloroform (Sigma-Aldrich) was added to all samples in a concentration of 200 µL/mL RNA-Bee. Total RNA from synovium explants was purified by using RNeasy Micro Kit (Qiagen, Hilden, Germany) according to the manufacturer’s instructions. RNA yield and purity of samples were determined using NanoDrop ND1000 UV-Vis Spectrophotometer (Isogen Life Science, Veldzicht, The Netherlands) at 260/280 nm. Purified RNA was reverse-transcribed to complementary DNA (cDNA) using the RevertAid First Strand cDNA Synthesis Kit (Fermentas GmbH, Leon-Rot, Germany) according to the manufacturer’s instructions. Quantitative real-time polymerase chain reaction (qRT-PCR) was performed with 10 µL of the sample with TaqMan^®^ Universal PCR Master Mix (Applied Biosystems) or Mastermix Plus for SYBR^®^ Green I (Eurogentec) using the CFX96 Touch™ Real-Time PCR Detection System (Biorad, Hercules, California, USA). Expression of genes encoding for glyceraldehyde-3-phosphate dehydrogenase (*GAPDH*), hypoxanthine phosphoribosyltransferase 1 (*HPRT*), ubiquitin C (*UBC*), tumor necrosis factor-alpha (*TNFA*), interleukin-1β (*IL1B*), IL6 (*IL6*), chemokine (C-C motif) ligand 18 (*CCL18*), mannose receptor C-type 1 (*MRC1*), coding for CD206, and CD163 (*CD163*) was assessed with qRT-PCR (**Table 1**). The geometric mean of the genes *GAPDH*, *HPRT*, and *UBC* was used to calculate the BestKeeper index (BKI). There were no significant differences between the conditions: 0.01% DMSO and treatment with medication did not interfere with best housekeeper gene levels (data not shown). The relative gene expression of genes of interest was calculated according to the 2^−ΔCT^ formula.

**Table 1.** List of Primers Used to Detect mRNA Levels by Quantitative Real-Time Polymerase Chain Reaction.

Gene Name	Forward	Reverse	Probe
*GAPDH*	GTCAACGGATTTGGTCGTATTGGG	TGCCATGGGTGGAATCATATTGG	CGCCCAATACGACCAAATCCGTTGAC
*HPRT*	TATGGACAGGACTGAACGTCTTG	CACACAGAGGGCTACAATGTG	AGATGTGATGAAGGAGATGGGAGGCCA
*UBC*	ATTTGGGTCGCGGTTCTTG	TGCCTTGACATTCTCGATGGT	NA
*TNFA*	GCCGCATCGCCGTCTCCTAC	GCGCTGAGTCGGTCACCCT	NA
*IL1B*	CTAAACA-GATGAAGTGCTCCT	TAGCTGGATGCCGCCAT	NA
*IL6*	TCGAGCCCACCGGGAACGAA	GCAGGGAAGGCAGCAGGCAA	NA
*CCL18*	GCACCATGGCCCTCTGCTCC	GGGCACTGGGGGCTGGTTTC	NA
*MRC1*	TGGCCGTATGCCGGTCACTGTTA	ACTTGTGAGGTCACCGCCTTCCT	NA
*CD163*	GCAATGGGGTGGACTTACCT	TCACCATGCTTCACTTCAACAC	NA

### Fluorescence Activated Cell Sorting (FACS) of Macrophage Phenotypes

Synovial explants were digested for 3 hours at 37°C in Hanks’ Balanced Salt solution (Gibco) with Ca2+ and Mg2+, containing 2 mg/mL Collagenase IV (Gibco) and 0.2 mg/mL Dispase II (Roche, Penzberg, Upper Bavaria, Germany). At the end of digestion FCS was added to a final concentration of 5% and the cell suspension was filtered first using a 100 µm filter, and then twice through a 40 µm filter. Cells were then centrifuged for 8 minutes at 250*g*, resuspended in FACSflow (#342003 BD Biosciences) and counted. Approximately 200,000 cells were stained for each condition. Cells were resuspended in 40 μL of FACSflow and incubated for 15 minutes at room temperature in the dark with surface antibody (Ab) solutions containing mixes of the following Abs; CD14 (APC-H7, #561384), CD206 (FITC, #551135), CD163 (PerCP-Cy™5.5, #563887), CD80 (PECy™7, #561135), and CD86 (PE, #560957) all purchased from BD Biosciences with dilutions according to manufacturer’s instructions. Cells were fixed in 1.8% paraformaldehyde for 20 minutes in the dark. Subsequently, cells were resuspended in FACSflow and analyzed with FACSJazz (BD Biosciences) and FlowJo Software (Tree Star, Palo Alto, CA, USA).

### Monocyte Isolation, Polarization, and Modulation of Macrophages

Monocytes were obtained from 4 healthy male buffy coat donors (Sanquin, Rotterdam) as previously described.^29^ After Ficoll (GE Healthcare, Little Chalfont, UK) density gradient centrifugation, peripheral blood mononuclear cells (PBMCs) were labeled with anti-CD14 magnetic beads (MACS; Miltenyi, Bergisch Gladbach, Germany) and isolated by MACS. Monocytes were seeded at a density of 500,000 monocytes/cm^2^ in X-VIVO™ medium (Lonza, Verviers, Belgium) supplemented with 20% heat-inactivated fetal calf serum (FCS; Lonza), 50 µg/mL gentamicin (Gibco) and 1.5 µg/mL amphotericin B (Gibco). Monocytes were polarized with 10 ng/mL tumor necrosis factor-α (TNF-α; PeproTech) and interferon-γ (INF-γ; PeproTech) toward a pro-inflammatory M(TNFα+IFNγ) macrophage phenotype, with 10 ng/mL interleukin-4 (IL-4; PeproTech) toward a repair M(IL-4) phenotype or with interleukin-10 (IL-10; PeproTech) toward an anti-inflammatory M(IL-10) phenotype. After 72 hours the medium with the stimuli was removed and refreshed for another 24 hours with the addition of 1 µM TAA (Sigma-Aldrich) to modulate their phenotype or 0.01% DMSO as vehicle control. After this polarization period, the different macrophage phenotypes were cultured for an additional 24 hours in DMEM-LG (Gibco) supplemented with 1% ITS Premix (Corning), 50 mg/mL gentamicin (Gibco), 1.5 mg/mL amphotericin B (Fungizone; Gibco) in the presence of 1 µM TAA (Sigma-Aldrich) or 0.01% DMSO as a control. We decided on this setup to mimic the situation in the joint where inflammatory factors that polarize macrophages will be present initially when TAA is added. For these last 24 hours we removed the polarization stimuli since they would have an effect on BMSC migration, but kept TAA after we confirmed that the selected dose of TAA did not affect migration. The macrophage conditioned medium (MCM) was harvested, centrifuged for 8 minutes at 300*g* and stored at −80°C until used in the Boyden chamber migration assay (**Fig. 1**).

### Statistical Analysis

Per each group and donor, 3 to 6 biological replicates have been included, each of them measured in technical duplicates. The values of the technical duplicates were averaged before statistical analysis was applied. Gene expression, FACS, and Boyden chamber migration assay data were analyzed using a hierarchical statistical model, the linear mixed model. The different conditions were considered a fixed parameter and the donors (experiments), with the related biological replicates, as a random factor. Dose-response Boyden chamber migration assay data were analyzed with a Kruskal-Wallis test with Dunn’s multiple comparison test to analyze the different doses. Correlation coefficients were calculated using a Spearmen rho test for nonparametric data and a Pearson rho test for normally distributed data. All statistical analysis included 2-tailed tests. A *P* value of <.05 was considered to indicate statistical significance. SPSS statistics package version 27.0 for MAC (SPSS Inc., Chicago, IL, USA) was used for all analyses.

## Results

### Synovium CM Stimulated MSC Migration in a Dose- and Donor-Dependent Way

**Figure 2.**
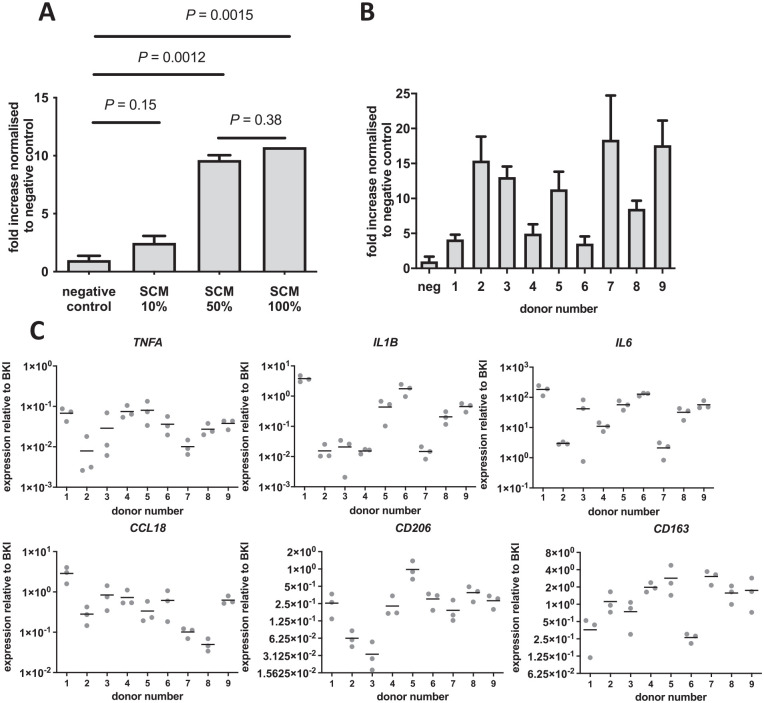
Synovium conditioned medium resulted in a dose- and donor-dependent increase in MSC migration. (**A**) MSC migration in response to different SCM doses from 3 pooled donors normalized to unconditioned medium (=negative control). The average number of cells that migrated in the control condition was 279. (**B**) MSC migration in response to 9 different SCM donors. Each bar represents the fold increase normalized to the negative control (unconditioned DMEM-LG 1% ITS) + SD. The average number of cells that migrated in the control condition was 233. n = 9 donors in quintuplicate. (**C**) Gene expression level of *TNFA*, *IL1B*, *IL6*, *CCL18*, *CD206*, and *CD163* relative to the BKI (*GAPDH, HPRT, UBC*). Each dot represents 1 of 3 explants from 9 synovium donors. MSC = mesenchymal stromal cell; SCM = synovium conditioned medium; DMEM-LG = Dulbecco’s Modified Eagle Medium, low glucose; ITS = Insulin-Transferrin-Selenium; TNFA = tumor necrosis factor-alpha; BKI = BestKeeper index; GAPDH = glyceraldehyde-3-phosphate dehydrogenase; HPRT = hypoxanthine phosphoribosyltransferase 1; UBC = ubiquitin C.

The effect of factors secreted by synovium on the migration of MSCs was investigated by using the 2CM of synovium explants (SCM). MSC migration was significantly stimulated by both 50% and 100% SCM compared to the unconditioned medium, whereas 10% SCM failed to stimulate migration (**Fig. 2A**). Since there was no incremental difference between 50% and 100% CM (*P* = 0.38) (**Fig. 2A**), 50% was used for all further experiments described in this article. SCM resulted in a donor-dependent increase of MSC migration that varied between 3.5-fold and 18.4-fold (**Fig. 2B**). To evaluate whether this difference between synovium donors was dependent on the level of inflammation, we analyzed inflammation-related gene expression levels. Similar to the migration of MSCs in response to SCM, the level of inflammation in the synovium explants varied between donors. The expression levels of *TNFA* (r = −0.46, *P* = 0.21), *IL*1B (r = −0.55, *P* = 0.13), *IL6* (r = −0.63, *P* = 0.076), *CCL18* (r = −0.37, *P* = 0.33), *CD206* (r = −0.40, *P* = 0.29), and *CD163* (r = 0.60, *P* = 0.09) were not correlated the number of MSCs for these individual cytokines (**Fig. 2C**).

### Inflammation of the Synovial Explants Was Reduced in Response to Anti-Inflammatory Treatment and This Increased MSC Migration

**Figure 3.**
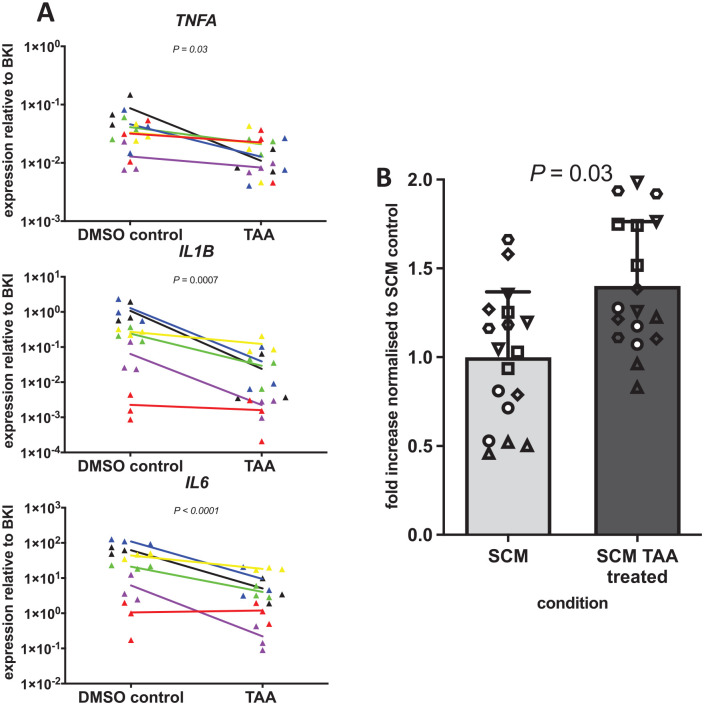
Reduced synovial inflammation increased MSC migration. (**A**) Gene expression level of *TNFA*, *IL1B*, and *IL6* relative to the BKI (*GAPDH*, *HPRT*, *UBC*). Each triangle represents 1 sample of 3 explants. Each color represents a different donor (n = 6 in total). The line between the dots represents the difference between the mean of the biological triplicates for the conditions without and with 1 µM TAA for each donor. (**B**) MSC migration in response to conditioned medium from synovium explants modulated without and with 1 µM TAA. To normalize the data of the different experiments, the average of the conditioned control condition (SCM) in each experiment was set to 1. The average number of cells that migrated in the control condition was 3,783. The bars represent the mean increase + SD. n = 6 synovium donors in triplicate. Each synovium donor is represented by a different symbol. MSC = bone marrow–derived mesenchymal stromal cell; TNFA = tumor necrosis factor-alpha; BKI = BestKeeper index; GAPDH = glyceraldehyde-3-phosphate dehydrogenase; HPRT = hypoxanthine phosphoribosyltransferase 1; UBC = ubiquitin C; TAA = triamcinolone acetonide; SCM = synovium conditioned medium; DMSO = dimethyl sulfoxide.

To further study the relation between synovial inflammation and the number of migrating MSCs, TAA was used to investigate how modulation of inflammation would influence MSC migration. TAA’s efficiency was confirmed by a significant reduction of the expression of the inflammation-related genes *TNFA*, *IL1B*, and *IL6* in the synovium (**Fig. 3A**). Then, we investigated MSC migration in response to CM from synovium modulated with TAA. First, we showed that the presence of TAA did not influence the effect of SCM on MSC migration, independent of the dose of both SCM and TAA (Suppl. Fig. S1). We therefore kept TAA in the CM for all further experiments. MSC migration was higher in response to medium conditioned by synovium modulated with TAA compared to unmodulated synovium (Suppl. Fig. S2), resulting on average in a 1.5-fold increase in migration (95% confidence interval (CI) = 0.04-0.94, *P* = 0.03) (**Fig. 3B**).

### Modulation of Synovial Inflammation with TAA Increased MSC Migration Through Repair- and Anti-Inflammatory Macrophages

**Figure 4.**
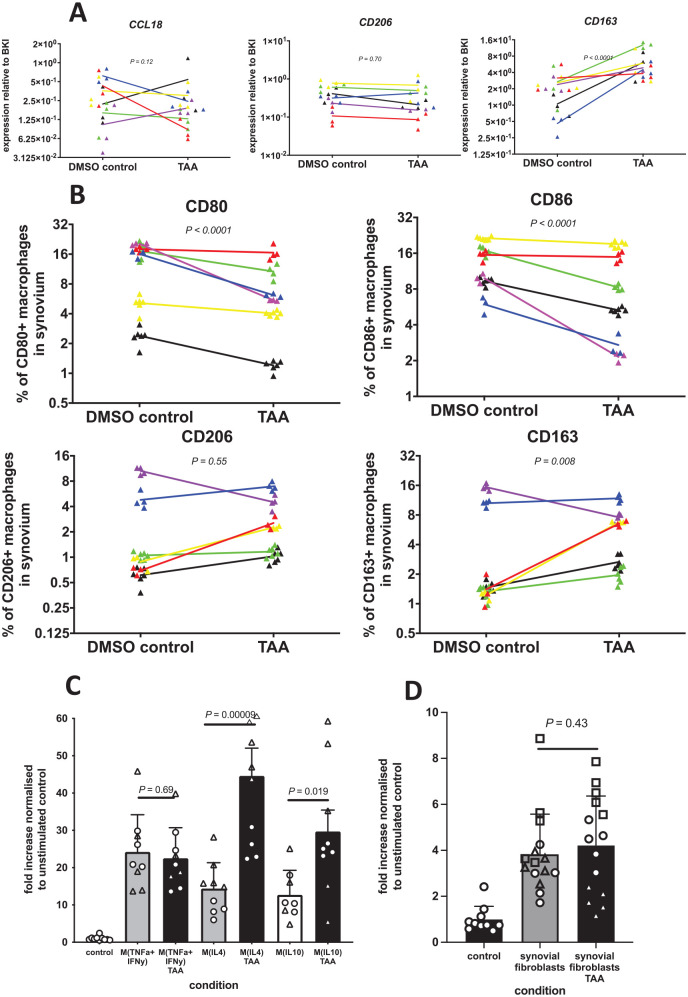
Modulation of synovial inflammation by TAA increased MSC migration through repair M(IL4) and anti-inflammatory M(IL10) macrophages treated with TAA. (**A**) Expression of repair/anti-inflammatory macrophage-related genes *CCL18*, *CD206*, and *CD163* is shown relative to the BKI. Each dot represents 1 sample of 3 explants. Each color represents a different synovium donor (n = 6). (**B**) The percentages of pro-inflammatory (CD80 and CD86) and anti-inflammatory surface markers (CD163 and CD206) among CD14+ macrophages in the synovium are shown. Each dot represents 1 sample of 5 explants. Each color represents a separate donor (n = 6). The lines indicate the difference in the mean of the explants with and without 1 µM TAA for each individual donor. (**C**) MSC migration in response to conditioned medium from pro-inflammatory M(TNFα+IFNγ), repair M(IL4) macrophages, and anti-inflammatory M(IL10) macrophages compared to conditioned medium from 1 µM TAA modulated macrophage phenotypes. The average number of cells that migrated in the control condition was 142. *n* = 2 batches of 2 pooled monocyte donors (4 donors in total), first batch in quadruplicate, second batch in quintuplicate. Each batch of 2 donors is represented by a different symbol. (**D**) MSC migration in response to conditioned medium from synovial fibroblasts and conditioned medium from 1 µM TAA modulated synovial fibroblasts. The average number of cells that migrated in the control condition was 150. Each bar represents the fold increase normalized to the negative control (unconditioned DMEM-LG 1% ITS) + SD. *n* = 3 synovial fibroblast donors in quintuplicate. Each synovial fibroblast donor is represented by a different symbol. TAA = triamcionolone acetonide; MSC = bone marrow–derived mesenchymal stromal cell; BKI = BestKeeper index; DMEM-LG = Dulbecco’s Modified Eagle Medium, low glucose; ITS = Insulin-Transferrin-Selenium; DMSO = Dimethyl sulfoxide.

To further investigate the contribution of the cell types present in the synovium, we investigated whether TAA alters the phenotype of the macrophages. Next to the downregulation of *TNFA*, *IL1B* and *IL6*, pro-inflammatory macrophage markers (**Fig. 3A**), *CCL18* and *CD206*, 2 repair macrophage markers, were not affected by TAA treatment (**Fig. 4A**) and the anti-inflammatory macrophage marker, *CD163*, was increased (**Fig. 4A**). We then evaluated the effect of TAA on the composition of macrophages in the synovium and observed that TAA treatment resulted in a significantly lower percentage of CD14+/CD80+ (95% CI = −7.63 to −4.48, *P* < 0.0001) and CD14+/CD86+ (95% CI = −5.22 to −3.57, *P* < 0.0001) pro-inflammatory macrophages in the synovium. On the contrary, the percentage of CD14+/CD163+ (95% CI = 0.42-2.64, *P* = 0.008) anti-inflammatory macrophages was significantly higher in synovial explants modulated with TAA (**Fig. 4B**). This indicates that treating synovial samples with TAA decreased pro-inflammatory macrophages and increased anti-inflammatory macrophages.

To investigate whether this shift in macrophage phenotype could be responsible for the increase in MSC migration in response to synovium explants treated with TAA, we analyzed MSC migration in response to macrophage CM. CM from all macrophage phenotypes, derived from peripheral blood monocytes, stimulated MSC migration (**Fig. 4C**). Then we treated macrophages with different phenotypes with TAA and evaluated the effect of their CM on MSC migration. CM from repair M(IL4) and anti-inflammatory M(IL10) macrophages treated with TAA increased MSC migration 2- to 3-fold compared to untreated macrophages (95% CI = 18.10-42.28, *P* = 0.00009 and 95% CI = 3.19-30.80, *P* = 0.019) (**Fig. 4C**). Modulation of pro-inflammatory macrophages M(TNFα+IFNγ) with TAA did not result in a change in MSC migration (**Fig. 4C**). In addition, modulation of synovial fibroblasts (the other main cell type of synovium) with TAA did not increase MSC migration (**Fig. 4D**). These results indicate that the increase in MSC migration in modulated synovial samples is probably through the specific role of repair M(IL4) and anti-inflammatory M(IL10) macrophages.

## Discussion

The first step in endogenous cartilage repair strategies is the recruitment of progenitor cells that can generate the repair tissue. In joints with a cartilage defect, the intra-articular joint environment is often disturbed and characterized by synovial inflammation.^30^ In this study, we assessed how modulation of synovial inflammation influences MSC migration and elucidated the role of different cell types present in the inflamed synovium. We found that reducing synovial inflammation with TAA increased MSC migration *in vitro*, and that this stimulatory effect appears to be mediated by repair- and anti-inflammatory macrophages.

In our study, conditioned media from all macrophage phenotypes dose-dependently increased MSC migration, indicating that the macrophages secrete factors that can stimulate MSC migration. Since macrophages are the most abundant cell type in the synovium, they play an important role in the disturbed homeostasis in a joint with a cartilage defect. To drive the joint environment to a more favorable cartilage repair environment, we modulated the macrophages with TAA. To our knowledge, this is the first study that shows how modulation of synovial inflammation may enhance progenitor cell recruitment to the defect site, with a proposed key role for anti-inflammatory macrophages. The observation that TAA increases repair- and anti-inflammatory macrophages present in the synovium is in line with a study showing that the phenotype of macrophages in the synovium can be modulated toward an anti-inflammatory phenotype upon using dexamethasone^27^ and a study on an acute lung injury mouse model that showed that methylprednisolone reduced the number of pro-inflammatory macrophages and increased the number of anti-inflammatory and repair macrophages.^31^ Since these studies used a different glucocorticoid, this indicates that the overall effect of glucocorticoids on macrophage phenotype is similar.

An increase in anti-inflammatory and repair macrophages can occur through a change in the polarization state of pro-inflammatory macrophages into a more anti-inflammatory phenotype. We observed a decrease in the percentage of pro-inflammatory macrophages and at the same time an increase in anti-inflammatory macrophages, indicating that the polarization state of the macrophages might have changed. Our results are supported by a study that reported a decrease in CD80+ cells and an increase in CD163+ cells upon treatment with TAA.^32^ In addition, glucocorticoids may stimulate the differentiation from naïve monocytes into an anti-inflammatory macrophage phenotype.^33^ We cannot discriminate between the mechanisms that could be responsible for the increase in anti-inflammatory macrophages in the synovium and decrease in pro-inflammatory macrophages, and it will most likely be a combination of these two. Whether these mechanisms play a role when glucocorticoids are applied to the joint *in vivo* remains to be investigated. Moreover, glucocorticoids have been shown to inhibit the migration of pro-inflammatory macrophages and stimulate the migration of anti-inflammatory macrophages,^33,34^ which can play an additional role when glucocorticoids are applied *in vivo*.

Mesenchymal stromal cells have been shown to be attracted to sites of inflammation, in studies ranging from skin wounds to tumor environments.^35,36^ Many of the factors present in a joint with a cartilage defect have been widely investigated on an individual basis and overall have a stimulatory effect on the migration capacity of MSCs.^19^ However, joint inflammation cannot be attributed to a single factor, but is much more complex and most likely a “cocktail” of factors that contain both pro-inflammatory and anti-inflammatory factors. Therefore, it might not be the best option to focus on the inhibition or stimulation of one specific cytokine, but rather on modulating this “cocktail” of factors. By using TAA we demonstrated to inhibit multiple pro-inflammatory markers and stimulated anti-inflammatory markers. This effect is likely to alter the “cocktail” of factors, which is supported by a study showing that intra-articular glucocorticoid treatment reduced synovial cell infiltration and pro-inflammatory cytokine expression in chronic arthritis.^37^ The change in pro-inflammatory and anti-inflammatory factors could contribute to a more favorable joint environment that in turn might attract more MSCs from the bone marrow.

TAA itself had no direct effect on the migration of MSCs from bone marrow. These data are at odds with the previous findings that described TAA inhibited cell migration of tenocytes and outgrowth of ligament and synovial capsule cells.^38,39^ However, in those studies, the exposure to TAA varied from 4 to 14 days and was thereby much longer than the 16 hours in our study and the shorter exposure to TAA of 4 days was shown to give less inhibition of cell migration.^38^ Nevertheless, this knowledge should be considered when using TAA in a clinical setting, since after intra-articular injection TAA could be detected up to 15 days.^40^ Moreover, a recent case report suggested that high dose of steroids injected intra-articulary could transiently compromise the chondrogenic capacity of MSCs from synovium.^41^ Also, there is an ongoing debate among the effect TAA has on the articular cartilage. The current evidence is conflicting and shows both beneficial and detrimental effects on the cartilage.^42^ Therefore, it might be of value to explore whether other drugs can also successfully reduce inflammation and improve MSC migration.

After stimulation of migration of bone marrow–derived MSCs by surgically making holes from the cartilage defect into the subchondral bone, chondrogenesis and subsequent cartilage tissue formation should take place in endogenous cartilage repair procedures.^43^ Progenitor cells from synovium can also play a role in cartilage repair too.^44^ It was shown that bone marrow–derived MSCs migrate roughly 3x more than synovium-derived MSCs in response to 10% FCS. This difference in migration capacity indicates that bone marrow–derived MSCs might be more important in migration toward the defect site than MSCs from the synovium;^45^ future studies should explore whether the difference in migration capacity also exists in response to synovium secreted factors. Pro-inflammatory factors are known to inhibit the chondrogenic capacity of MSCs *in vitro.*^46,47^ Furthermore, the CM obtained from inflamed osteoarthritic synovium reduced the chondrogenic capacity of MSCs, and this effect was assigned predominantly to pro-inflammatory macrophages in the synovium.^48^ The transition from pro-inflammatory macrophages toward anti-inflammatory macrophages might, besides increasing the migration of MSCs, also be beneficial for the cartilage formation capacity. Moreover, anti-inflammatory/repair macrophages might even enhance the cartilage-forming capacity of MSCs.^14^ This implicates that modulating pro-inflammatory macrophages toward a more anti-inflammatory phenotype could be a promising tool to improve the success of endogenous cartilage repair strategies.

## Supplemental Material

sj-docx-1-car-10.1177_19476035221085136 – Supplemental material for Modulation of Inflamed Synovium Improves Migration of Mesenchymal Stromal Cells in Vitro Through Anti-Inflammatory Macrophages Click here for additional data file. Supplemental material, sj-docx-1-car-10.1177_19476035221085136 for Modulation of Inflamed Synovium Improves Migration of Mesenchymal Stromal Cells in Vitro Through Anti-Inflammatory Macrophages by Marinus A. Wesdorp, Yvonne M. Bastiaansen-Jenniskens, Serdar Capar, Jan A.N. Verhaar, R. Narcisi and Gerjo J.V.M. Van Osch in CARTILAGE
