# Supplementary material for: Modulation of Inflamed Synovium Improves Migration of Mesenchymal Stromal Cells in Vitro Through Anti-Inflammatory Macrophages
Source: Cartilage. 2022 Mar 19;13(1):19476035221085136. doi: 10.1177/19476035221085136 (PMC9137323; doi:10.1177/19476035221085136)
Supplement: sj-docx-1-car-10.1177_19476035221085136 – Supplemental material for Modulation of Inflamed Synovium Improves Migration of Mesenchymal Stromal Cells in Vitro Through Anti-Inflammatory Macrophages [file sj-docx-1-car-10.1177_19476035221085136.docx]

**Table S1. List of synovium donors**

| **Donor** | **Sex** | **Age (years)** |
| --- | --- | --- |
| 1 | F | 57 |
| 2 | F | 73 |
| 3 | F | 69 |
| 4 | M | 72 |
| 5 | M | 53 |
| 6 | M | 68 |
| 7 | M | 62 |
| 8 | M | 50 |
| 9 | F | 63 |

**Table S2. List of MSC donors**

| **Donor** | **Sex** | **Age (years)** |
| --- | --- | --- |
| 1 | M | 23 |
| 2 | F | 69 |
| 3 | M | 71 |

**Supplementary Figure 1. The presence of TAA in SCM did not affect MSC migration**

MSC migration in response to 3 different SCM donors. Each bar represents the fold increase normalized to the negative control (unconditioned DMEM-LG 1% ITS) + SD. Abbreviations: SCM, synovium conditioned medium; TAA, triamcinolone acetonide; MSC, mesenchymal stromal cell.

**Supplementary Figure 2. MSC migration increased when synovial explants were treated with TAA.**

A) MSC migration in response to 6 different SCM donors compared to conditioned medium from the same donor modulated with 1µM TAA. The bars represent the mean increase + SD. n = 6 donors in triplicate. Abbreviations: SCM, synovium conditioned medium; TAA, triamcinolone acetonide; MSC, mesenchymal stromal cell.
